# Key Findings from the European Men-Who-Have-Sex-With-Men Internet Survey in Greece

**DOI:** 10.3390/epidemiologia2010010

**Published:** 2021-03-17

**Authors:** Katerina Pantavou, Georgios Tsiakalakis, Sophocles Chanos, Georgios Polkas, Georgios Papageorgiou, Nicolaos Dedes, Axel J. Schmidt, Georgios K. Nikolopoulos

**Affiliations:** 1 Medical School, University of Cyprus, 2029 Nicosia, Cyprus; pantavou.katerina@ucy.ac.cy; 2 Ath Checkpoint, 10554 Athens, Greece; gtsiakalakis@positivevoice.gr (G.T.); schanos@athcheckpoint.gr (S.C.); gpapageorgiou@positivevoice.gr (G.P.); nikos.dedes@positivevoice.gr (N.D.); 3 Thess Checkpoint, 54635 Thessaloniki, Greece; gpolkas@thesscheckpoint.gr; 4 Sigma Research, London School of Hygiene and Tropical Medicine, London WC1H 9SH, UK; axel.j.schmidt@emis-project.eu

**Keywords:** EMIS-2017, MSM, Greece, online survey, HIV, sexually transmitted infections, PrEP, PEP

## Abstract

The European Men-Who-Have-Sex-With-Men Internet Survey (EMIS-2017) is an international survey for men who have sex with men (MSM) designed to measure the level and distribution of four dimensions: (a) sexual health outcomes, (b) risk and precaution behaviors, (c) health promotion needs, and (d) coverage/uptake of interventions. The aim of the current work is to provide an overview of key demographics and findings for MSM in Greece covering the abovementioned dimensions of EMIS-2017, especially regarding HIV. Overall, 2909 men met the inclusion criteria for the analysis. The participants’ age ranged between 15 and 74 years old (median 35 years). According to the descriptive analysis, 14.4% of the participants reported moderate and 8.9% severe anxiety and depression. The self-reported HIV prevalence was 11%. A high number of participants had non-steady male partners (74%, *n* = 2153). The number of non-steady intercourse partners in the last 12 months was over two for about 61.5% (*n* = 1321) of the participants. A very small number of participants had ever tried to get pre-exposure prophylaxis (PrEP) (2.2%, *n* = 63), and 41.2% of the participants (*n* = 1199) were unaware of PrEP. About half of the participants (51.6%, *n* = 1501) did not know that vaccination against both hepatitis A and B viruses is recommended for MSM. The results of EMIS-2017 identify important needs and can help policy making and prevention planning.

## 1. Introduction

Men who have sex with men (MSM) are disproportionally affected by the human immunodeficiency virus (HIV) [1]. In Europe in particular, male-to-male sex is the predominant mode of HIV transmission [2]. Moreover, sexually transmitted infections (STIs) other than HIV, such as chlamydia, gonorrhea, and syphilis, continue to rise [3], affecting men who have sex with men (MSM) quite often [4]. In this context, the European Men-Who-Have-Sex-With-Men Internet Survey (EMIS-2017) was conducted aiming at collecting data useful for the planning of HIV and STI prevention and care programs, and the monitoring of national progress in this area [5]. 

EMIS-2017 was funded by the European Commission Health Programme 2014–2020 and supported by PlanetRomeo, European AIDS Treatment Group (EATG), Eurasian Coalition on Male Health (ECOM), European Centre for Disease Prevention and Control (ECDC), European Monitoring Centre for Drugs and Drug Addiction (EMCDDA), and European Commission (DG SANTE). EMIS-2017 was coordinated by Sigma Research at the London School of Hygiene and Tropical Medicine (LSHTM) in association with the Robert Koch Institute (RKI) in Berlin. The survey was conducted in 50 countries collecting data of 144,305 individual responses. More than 100,000 men in the European Union (EU) took part, in addition to more than 6000 in European Free Trade Association (EFTA) countries, and about 7000 in countries in the EU Enlargement Area or the European Neighborhood Policy. Moreover, 6000 MSM were recruited in Russia, 6000 in Canada, and 3500 in the Philippines [6].

The aim of this work is to describe key data collected in EMIS-2017 for Greece: sample population, morbidities and especially HIV infection, people’s behaviors that cause (risks) or protect them (precautions) from morbidities, opportunities, capabilities and motivations for risk and precaution behaviors, and actions of others that meet or undermine the needs of MSM.

## 2. Materials and Methods

EMIS-2017 (website: www.emis2017.eu (accessed on 11 March 2021)) was a multi-language, internet-based survey for MSM living in Europe or in a limited number of countries outside Europe. The survey was conducted in 33 languages using a multilingual online questionnaire [7] and used similar recruitment procedures across countries. It was promoted via international (and to a much smaller extent national) MSM dating apps and websites as well as via civil society organizations, such as Positive Voice. EMIS-2017 used a convenience sample following a non-probability sampling method and focusing on MSM who have access to the internet and/or use gay dating apps.

EMIS-2017 was approved by the ethics committee of the London School of Hygiene and Tropical Medicine (University of London). All participants in EMIS-2017 were asked to confirm that they consent to participate. Details on the design and methods used in EMIS-2017 can be found in the article of Weatherburn and colleagues [8].

EMIS-2017 was carried out simultaneously in 50 countries, including Greece, from 18 October 2017 to 31 January 2018. Eligible participants in the Greece survey were men: (a) living in Greece, (b) at or over the age of homosexual consent (i.e., 15 years), (c) who are sexually attracted to men and/or had sex with men, and (d) who reported that they understand the nature and purpose of the study and consent to take part. The participants were asked to self-complete a questionnaire in the language of their choice, and they could withdraw at any time. 

### 2.1. Study Area

Greece is a Mediterranean country in Southeastern Europe. The population is approximately 10.8 million (males, 5.3 million) based on the 2011 census [9]. The nation’s capital, Athens, is the largest and most populous area of the country with 3.8 million residents [9]. 

Lesbian, gay, bisexual, and transgender (LGBT) culture is vibrant in several neighborhoods of Athens, in the second largest city of Greece, Thessaloniki, as well as on some Greek islands. Homosexual activity has been legal in Greece since 1951. Antidiscrimination laws in employment were enacted in 2005, and civil unions were legislated in 2015. The HIV epidemic has been mainly concentrated in MSM with the exception of 2011–2013, when an outbreak among people who inject drugs occurred (PWID) [10]. The cumulative number of HIV diagnoses (including AIDS cases) reported in Greece by 31 December 2017 was 16,669 (males: 82.84%, *n* = 13,808) [11]. After excluding cases with undocumented mode of HIV transmission, 57.8% (*n* = 8.076) of HIV diagnoses in Greece were in MSM [11]. 

### 2.2. Questionnaire

The questionnaire used in EMIS-2017 [7] was developed in English and then translated to Greek. A manual of the variables in the questionnaire [12] is also available at the EMIS-2017 website. Some slang terms were included in parentheses in order to increase responses to questions regarding socially “undesirable” behavior [4]. The EMIS-2017 questionnaire was based on a previous version of the survey, EMIS-2010 [13].

Questionnaire items were divided in five sections: (a) “Demographics”, (b) “Morbidities”, (c) “Behaviours”, (d) “Needs”, and (e) “Interventions”. The “Demographics” section collects information on key characteristics of the participants (i.e., gender identity, sex at birth, age, country of birth, length of residents’ stay in a given country, education, employment, sexual attraction, sexual identity, and outness). The “Morbidities” section includes items about both psychological and physical health and focuses on mental health problems among MSM (i.e., anxiety and depression, suicidal ideation, alcohol dependency) and on sexually transmitted infections (i.e., HIV, hepatitis, syphilis, gonorrhea chlamydia, and anogenital warts). Two measures of mental health were used, the Patient Health Questionaire-4 (PHQ-4) for anxiety and depression [14] and the four CAGE questions for alcohol use [15]. The “Behaviours” section collects information on activities of participants that could contribute to or detract from morbidities. It includes items about sexual health risk (having sex, taking drugs, and doing them together) and precaution behavior (taking antiretroviral drugs or HIV chemoprophylaxis and being vaccinated). The “Needs” section focuses on commonly unmet sexual health needs of the MSM population that could inform the design of interventions. The Intervention section refers to actions of people that meet (positive intervention) or undermine (negative intervention) the health promotion needs of MSM. Positive interventions include education, health and social services and negative interventions include homophobic legislation, exclusion, and abuse.

### 2.3. Statistical Analysis

Mean and median values and standard deviation and interquartile range were used to describe continuous variables. Frequencies and percentages were used for categorical variables. The statistical analysis was conducted in Stata v. 14 (Stata Corp., College Station, TX, USA).

## 3. Results

In EMIS-2017, 2943 individual cases were recorded as participants living in Greece. Of these, 34 failed to meet the following inclusion criteria: (a) identified as men or trans men (*n* = 24), (b) sexually attracted to men and/or having had sex with men (*n* = 8), and (c) at or over the age of homosexual consent in Greece (*n* = 2), leaving a sample of 2909 records qualified for the study.

### 3.1. Sample Description

Of the 2909 men included in the analysis, 1% (*n* = 28) identified as trans and 0.1% (*n* = 3) reported that the gender they were assigned at birth was female (decline to state, *n* = 1; not answered, *n* = 5, 0.2%) (Table 1). The age of the participants ranged between 15 and 74 years (Figure S1). The median age was 35 years (interquartile range: 27–42).

The majority of men (91%, *n* = 2646) reported that they were born in Greece (Table 1). Most men not born in Greece had been living in Greece for more than 10 years (73.2%, *n* = 191). More than 60% of the participants reported that they had spent over 6 years in full-time education since the age of 16 (60.2%, *n* = 1752), that they were employed full-time, part-time or were self-employed (65.9%, *n* = 1916), and that their feelings about their income these days were neither comfortable nor struggling or that they were living comfortably/really comfortably (72.4%, *n* = 2160) (Table S1). 

Almost three quarters of men thought of themselves as gay or homosexual (72.2%, *n* = 2099) and 15.1% (*n* = 440) as bisexual (Table S2). The percentage of men that were out about their sexual attraction to men was limited; 32.7% (*n* = 941) reported that few people know and 12.9% (*n* = 371) reported that no one knows that they are attracted to men (Table S2). About one third of men currently had a steady partner (29.8%, *n* = 867), and 15.5% (*n* = 450) reported that they had never had a steady relationship (Table S3). Paying for sex (20.3%, *n* = 590) was more common than being paid for sex (11.3%, *n* = 328) (Table S4). 

### 3.2. Morbidities and Health Outcomes 

#### 3.2.1. Mental Health

Anxiety and depression of 23.3% (*n* = 679) of participants was “moderate” (14.4%, *n* = 420) or “severe” (8.9%, *n* = 259) (Table 2 and Table S5). The most common bothering problem was feeling nervous, anxious or on the edge (46.8%, *n* = 1361; Table S5). Over the last 2 weeks, 18.3% (*n* = 533) of the respondents had been bothered by thoughts that they would be better off dead, or of hurting themselves in some way (Table 2). The CAGE screening tool [15] showed that 289 (9.9%) of the men in the survey met the criteria for potential alcohol dependency (Table 2). Most positive responses on alcohol dependency were reported on the questionnaire item asking participants whether they had tried to cut down on their drinking (23.3%, *n* = 602; Table S6).

#### 3.2.2. Sexually Transmitted Infections

**Figure 1:**
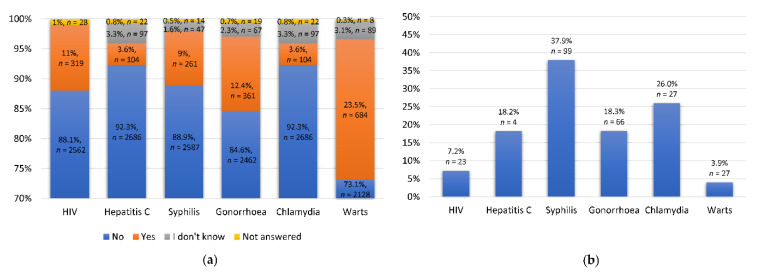
Diagnoses of sexually transmitted infections. The participants self-reported (**a**) whether they had ever been diagnosed with HIV, hepatitis C, syphilis, gonorrhea, chlamydia or anogenital warts and (**b**) whether they had been diagnosed with HIV, syphilis, gonorrhea, chlamydia or whether they were first diagnosed with hepatitis C or anogenital warts within the last 12 months.

Overall, 11% of the participants reported an HIV diagnosis (Figure 1a). Of those, 7.2% (*n* = 23) had been diagnosed within the last 12 months (Figure 1b). Moreover, most of them had an undetectable viral load (73%, *n* = 233). The most common infection was that with human papillomavirus (HPV). About 23.5% (*n* = 684) of the participants reported that they had been diagnosed with anogenital warts in their lifetime (Figure 1a), and 3.9% (*n* = 27) of them had first been diagnosed within the last 12 months (Figure 1b). Diagnoses of syphilis and gonorrhea were reported by 9% (*n* = 261) and 12.4% (*n* = 361) of the participants, respectively. Of these men, 37.9% (*n* = 99) had been diagnosed with syphilis and 18.3% (*n* = 66) with gonorrhea in the last 12 months. The most common STI within the last 12 months was syphilis (Figure 1b).

### 3.3. Risk and Precaution Behavior

#### 3.3.1. Sex and Drugs

There were men participating in EMIS-2017 who had never had sexual contact or intercourse with another man. Table S7 shows that 2.9% (*n* = 85) of men had never had any kind of sex with a man and 7.3% (*n* = 212) had never had any intercourse. One in ten reported that the first time they had any sex with another male they were 13 years old or younger than 13 (11%, *n* = 310), and 27.5% (*n* = 775) when they were between 14 and 17 years old. About 74% (*n* = 2153) of the participants had non-steady male partners and 8.1% (*n* = 153) never used or used seldom condoms when they had intercourse with non-steady male partners. About 61.5% (*n* = 1321) reported 3 or more non-steady male partners in the last 12 months (Table S7). 

The most commonly used substance was alcohol; 91.2% (*n* = 2653) of the respondents had consumed alcohol in their lifetime and 59.7% (*n* = 1736) within the last seven days (Figure S2). Alcohol consumption was followed by use of tobacco products; 68.7% (*n* = 2003) had used tobacco in their lifetime. Another two substances used by about 30% of the participants were poppers and cannabis. Sex under intoxication was reported by 40.9% (*n* = 1098) of men in the last 12 months (Table S8). 

#### 3.3.2. Antiretroviral Treatment (ART), HIV Chemoprophylaxis, and Vaccination

Among men who had been diagnosed with HIV, 87.6% (*n* = 304) reported that they had taken antiretroviral treatment (ART) (Table 3). Of those, 98% (*n* = 298) were receiving ART at the time the survey was conducted (Table 3). Among men without an HIV diagnosis, 7.9% (*n* = 203) had tried to get post-exposure prophylaxis (PEP), of whom 66% (*n* = 134) had taken one or more than one course of pills (Table 3). Pre-exposure prophylaxis (PrEP) was reportedly taken by 0.9% of the participants (*n* = 27). 

Men who had completed the course of vaccination against hepatitis A comprised 36.8% (*n* = 1070) of participants and against hepatitis B 41.3% (*n* = 1202). The percentage of men who did not know whether they had been vaccinated against hepatitis A and B was 28.6% (*n* = 831) for hepatitis A and 28.4% (*n* = 825) for B (Table 4).

### 3.4. Needs

Considering self-efficacy for safe sex, 22.7% (*n* = 661) of men did not agree with the statement “The sex I have is always as safe as I want it to be”. Slightly fewer, 16.3% (*n* = 472), did not find it easy to say “no” to sex they do not want (Table S9). Self-reported knowledge of HIV was high. Over 93% of men reported that they already knew the facts included in the questionnaire items related to the general knowledge of HIV (Table S10), 83% about HIV transmission, and 90.4% about HIV testing. Fewer respondents knew that a person with HIV who is on effective treatment cannot pass their virus to someone else during sex (50%, *n* = 1466).

More participants reported that they had not heard of PrEP (41.2%, *n* = 1199) than that they had not heard of PEP (33.9%, *n* = 987) (Table 5A). The low level of knowledge of PEP and PrEP was also observed when men were asked to report whether they already know basic facts on PEP and PrEP (Table S11). The percentage of participants who already knew basic information related to PEP ranged from 39.8% to 56.4% and related to PrEP from 13% to 46.3%. Among men not diagnosed with HIV, 58.4% (*n* = 1496) reported that they were quite or very confident that they could get PEP if they thought they needed it (Table 5B). Moreover, 42.5% (*n* = 1089) responded that they would likely use PrEP if it was available and affordable to them (Table 5B).

The proportion of “already know” responses in the four out of five facts given for hepatitis ranged between 71.3% (*n* = 2073) and 89% (*n* = 2589) (Table S12). The most common unknown fact was that doctors recommend that MSM should be vaccinated against both hepatitis A and hepatitis B viruses (51.6%, *n* = 1501). The proportion of participants who could benefit from hepatitis A and B vaccination (i.e., men non-vaccinated against hepatitis or who have not completed the course of vaccinations or who were aware of their hepatitis vaccination status) and actually knew where to get vaccinated against hepatitis A or B was 36.3% (n =600) or 36.2% (n =551), respectively (Table S12). 

### 3.5. Interventions

In the last 12 months, 23.7% (*n* = 689) of the participants had been stared at or intimidated, 14.9% (*n* = 433) had had verbal insults directed to them, and 1.6% (*n* = 46) had been punched, hit, kicked, or beaten because someone knew or presumed they were attracted to men (Table S13). One third (38.6%, *n* = 1122) of the participants reported that they had received free condoms from gay or HIV organizations, saunas, clinics, bars or clubs in the last 12 months, and 71.2% (*n* = 2072) had bought condoms at a physical store (not online) (Table S14). 

About 11.4% (*n* = 330) of men reported that someone had ever spoken to them personally at a health service in Greece about PrEP (mainly at checkpoints, i.e., community centers for HIV and hepatitis B and C prevention and testing commonly collaborating with healthcare foundations and governmental organizations, *n* = 271), and 72.8% (*n* = 2120) reported that they had seen or heard any information about HIV or STIs specifically for MSM in the last 12 months (Table S15). One fifth of men who had been diagnosed with HIV (20.7%, *n* = 66) were dissatisfied or very dissatisfied with the support and information they had received when they were diagnosed HIV positive (Table S16). 

## 4. Discussion

This work provides an overview of the data collected in Greece in the context of EMIS-2017. The dataset for Greece consisted of 2909 participants with a median age of 35 years, mainly homosexual (72.2%), born in the country (91%), with over 6 years of full-time education since the age of 16 (60.2%), and a satisfactory self-perception of financial status (72.4%). These attributes were similar to those of participants from other countries involved in EMIS-2017 [5]. Outness and steady partnerships seem to be limited among MSM in Greece. In particular, less than half of the participants (39%) reported that people in their social network were aware of the participants’ attraction to men. This was lower than the reported percentage (58.8%) in the EMIS-2017 European report [5]. Steady partnerships of the MSM participants in Greece were less frequent (29.8%) than those reported by all participants in EMIS-2017 (38.6%) [5].

In terms of morbidities, anxiety and depression were more prevalent among EMIS-2017 participants in Greece than in Europe overall (61.6% versus 52%). Nevertheless, the severe anxiety/depression score was relatively lower (9%) compared to that of other countries (16%) [5]. HIV prevalence was relatively high (11%) given that HIV prevalence in EMIS-2017 countries ranged from 3% to 16%. Lower prevalence than in the entire EMIS-2017 population was found for syphilis (9% versus 14.2%), gonorrhea (12.4% versus 19.2%), and chlamydia (3.6% versus 13.9%). These findings are in accordance with the lower diagnosis rate of STIs in Greece compared to other European countries [16,17,18]. Of course, variability in testing policies and reporting patterns rather than different incidence rate of STIs may explain the abovementioned differences between Greece and other European countries. Regarding behaviors, there was a very low number of MSM in Greece who had ever tried to get (2.2%) or use (1%) PrEP, and about 40% of participants had been vaccinated against hepatitis A or B.

Many participants were unaware of important information with respect to HIV chemoprophylaxis and transmission, and regarding vaccination against hepatitis A and hepatitis B viruses. A significant percentage of participants were unaware of PEP (33.9%) or PrEP (41.2%) in accordance with other EMIS-2017 countries [5]. The relative low awareness for PrEP in Greece could be due to the fact that PrEP has not been administered in Greece yet. It was available only for participants of an implementation program named SOPHOCLES-P4G [19]. It is anticipated that the awareness of PEP or PrEP perhaps increased after EMIS-2017, mostly due to significant efforts of non-governmental organizations, and especially of those of HIV-positive people (Positive voice) and of checkpoints. 

More than half of the participants had experienced verbal violence at least once in their lives and one in five had faced physical violence. This high rate of verbal or physical aggression could perhaps explain, to some degree, why a substantial number of EMIS-2017 participants in Greece reported low outness and suicidal or self-destructive thoughts [20,21,22]. The vast majority of the participants (85.3%) reported that no-one had spoken to them about PrEP and when they received information, this was primarily through checkpoints. The latter finding highlights the important role of checkpoints in surveillance and prevention [23].

The results presented in this report are subject to the limitations of anonymous, self-completed, online, cross-sectional surveys as EMIS-2017. Data validity can be influenced by multiple submissions by one person and the uncertainty about whether the participant providing answers actually met the inclusion criteria of EMIS-2017. The absence of an interviewer may induce differences in interpretations of survey questions and answer options among the participants. Potential limited access and reduced ability to use technology are common disadvantages of online surveys that decrease the ability to generalize. Recall bias and social desirability bias also have to be taken into account. The sample was large but convenient as many participants were recruited from social media websites and online applications and were thus probably non-representative of all MSM. The comparison of the EMIS-2010 sample with a nationally representative sample of MSM in the UK showed that younger, gay MSM and those who are more sexually active were overrepresented [24]. Nevertheless, EMIS-2017 provides essential baseline information and gives the opportunity to assess important needs. In particular, EMIS-2017 can significantly benefit HIV and viral hepatitis prevention programs. 

## 5. Conclusions

The results could be generalized to MSM who are able to use technology, have access to the internet, and are probably users of social media websites and certain online applications. HIV prevalence in that population was considerable, and a significant number of participants were not aware of a major prevention tool such as PrEP, which, however, was not available in Greece at the time.

## Figures and Tables

**Table 1:** Current gender identity, sex at birth, country of birth (*n* = 2909), and length of residence in Greece (*n* = 261).

		*N*	%			*N*	%
**Gender Identity**	Man	2881	99.0	**Sex Assigned at Birth**	Male	2900	99.7
	Trans man	28	1.0		Female	3	0.1
**Country of Birth**	Greece	2646	91.0		Refuse to state	1	0.0
	Germany	72	2.5		Not answered	5	0.2
	Cyprus	38	1.3	**City of Residence**	Athens	1413	48.6
	Other	146	5.0		Thessaloniki	422	14.5
	Not answered	7	0.2		Other settings (population under 500,000)	900	30.9
**Years of Residence in Greece ^1^**	<1	10	3.8				
	1–5	43	16.4		Other settings (populations over 500,000)	36	1.2
	>5	203	77.9				
					Not answered	138	4.7

^1^ Question shown when the answer to question regarding the country of birth was other than Greece.

**Table 2:** Mental health, suicidal ideation, and alcohol dependency (*n* = 2909).

	**Normal** **(0–2)**	**Mild** **(3–5)**	**Moderate** **(6–8)**	**Severe** **(9–12)**	**Not Answered**
Anxiety and depression score (PHQ-4) ^1^	1116	1055	420	259	59
	(38.4)	(36.3)	(14.4)	(8.9)	(2.0)
Over the last 2 weeks, how often have you been bothered by thoughts that you would be better off dead, or of hurting yourself in some way?	**Not at all**	**Some days**	**More than half the days**	**Nearly every day**	**Not answered**
	2355	392	73	68	21
	(81.0)	(13.5)	(2.5)	(2.3)	(0.7)
Alcohol dependency (CAGE) ^2^	**Yes**	**No**	**Not answered**		
	289 (9.9)	2560 (88.0)	60 (2.1)		

^1^ Patient Health Questionaire-4 (PHQ-4) [14]; ^2^ CAGE questions for alcohol use [15].

**Table 3:** Seeking and uptake of antiretroviral treatment (ART), post-exposure prophylaxis (PEP), and pre-exposure prophylaxis (PrEP).

	*Ν*	%		*Ν*	%
**Have you ever taken antiretroviral treatment (sometimes known as ART or HAART (*n* = 347) ^1^**			**Are you currently taking antiretroviral treatment? (*n* = 304) ^2^**		
No	7	2.0	No	4	1.3
Yes	304	87.6	Yes	298	98.0
Don’t know	6	1.7	Not answered	2	0.7
Not answered	30	8.7			
**Have you ever tried to get PEP?** **(*n* = 2562) ^3^**			**Have you ever tried to get PrEP? (*n* = 2909)**		
No	2347	91.6	No	2833	97.4
Yes	203	7.9	Yes	63	2.2
Not answered	12	0.5	Not answered	13	0.4
**Have you ever taken PEP? (*n* = 203) ^4^**			**Have you ever taken PrEP? (*n* = 2909)**		
No, I could not get it	31	15.3	No	2853	98.1
No, I had the opportunity but decided not to take it	33	16.3	Yes, on a daily basis and I’m still taking it	13	0.4
Yes, I’ve taken one course of pills	110	54.2	Yes, on a daily basis but I’m no longer taking it	8	0.3
Yes, I’ve taken more than one course of pills	24	11.8	Yes, when I need it but not daily	6	0.2
I don’t know	5	2.5	I don’t know	7	0.2
			Not answered	22	0.8

^1^ Participants diagnosed with HIV; ^2^ participants who had ever taken antiretroviral treatment; ^3^ participants with a negative HIV test; ^4^ participants who had ever tried to get PEP.

**Table 4:** Hepatitis A and B vaccination status.

Have You Been Vaccinated against …?	Hepatitis A		Hepatitis B	
	*Ν*	%	*Ν*	%
No, because I’ve had hepatitis [A/B]	167	5.7	160	5.5
No, and I don’t know if I’m immune	634	21.8	512	17.6
No, I have chronic hepatitis B infection			9	0.3
Yes, and I completed the course	1070	36.8	1202	41.3
Yes, but I did not complete the course	186	6.4	160	5.5
Yes, but I did not respond to the vaccinations			24	0.8
I don’t know	831	28.6	825	28.4
Not answered	21	0.7	17	0.6
Total	2909	100	2909	100

**Table 5:** (A) Awareness of post-exposure prophylaxis (PEP) and pre-exposure prophylaxis (PrEP) (*n* = 2909) and (B) Confidence accessing PEP among men not diagnosed with HIV and certainty of intention to use PrEP (*n* = 2562).

		*Ν*	%		*Ν*	%
**A**	**Have you heard of …**	**PEP**			**PrEP**	
No		987	33.9		1199	41.2
Yes		1587	54.6		1409	48.4
Not sure		303	10.4		254	8.7
Not answered		32	1.1		47	1.6
**B**	**How confident are you that you could get PEP if you thought you needed it?**			**If PrEP was available and affordable to you, how likely would you be to use it?**		
Very confident		815	31.8	Very unlikely	460	18.0
Quite confident		681	26.6	Quite unlikely	238	9.3
A little confident		425	16.6	Not sure	771	30.1
Not at all confident		299	11.7	Quite likely	449	17.5
I don’t know		337	13.2	Very likely	640	25.0
Not answered		5	0.2	Not answered	4	0.2

## Data Availability

The data presented in this study may be available from the London School of Hygiene and Tropical Medicine upon reasonable request.

## Supplementary Materials

The following are available online at https://www.mdpi.com/2673-3986/2/1/10/s1, Figure S1: Age in years in the EMIS-2017 sample in Greece, Figure S2: Recency of using selected substances, Table S1: Education, current occupation/work status, and feelings about current income, Table S2: Sexual attraction, sexual identity, and outness, Table S3: Percentages of current partnerships, type of partnership, and when the last steady partnership broke up, Table S4: Buying and selling sex, Table S5: Mental health. Men were asked to report how often they had been bothered by the following problems, over the last two weeks, Table S6: Alcohol dependency, Table S7: Sex with non-steady male partners and condom use in the last 12 months, Table S8: Sex under intoxication, Table S9: Self-efficacy measures considering sex, Table S10: HIV transmission, testing and treatment knowledge, Table S11: Post-exposure prophylaxis (PEP) and pre-exposure prophylaxis (PrEP) knowledge, Table S12: Hepatitis knowledge and knowledge of where to get hepatitis A and B vaccinations among men who could benefit from hepatitis A and B vaccine, Table S13: Homophobic abuse: intimidation, insults, and violence, Table S14: Sources of condoms in the last 12 months and most common source, Table S15: Recency of seeing/hearing any information about HIV or STIs specifically for men who have sex with men, Table S16: Satisfaction with support and information received during HIV testing.
