# Supplementary material for: Key Findings from the European Men-Who-Have-Sex-With-Men Internet Survey in Greece
Source: Epidemiologia (Basel). 2021 Mar 17;2(1):114–23. doi: 10.3390/epidemiologia2010010 (PMC9620921; doi:10.3390/epidemiologia2010010)
Supplement: Supplementary file 1 [file epidemiologia-02-00010-s001.pdf]

Supplementary material: Key Findings from the European Men-Who-Have-Sex-With-Men Internet Survey in Greece

Katerina Pantavou, Georgios Tsiakalakis, Sophocles Chanos, Georgios Polkas, Georgios Papageorgiou, Nicolaos Dedes, Axel J. Schmidt and Georgios K. Nikolopoulos

Table S1. Education, current occupation/work status, and feelings about current income.

		N	%
**How many years have you spent in full-time education since the age of 16?**	None	89	3.1
	1 to 5	772	26.5
	6 to 10	1298	44.6
	Over 10	454	15.6
	Not answered	296	10.2
	Total	2909	100
**Which of the following best describes your current occupation?**	Employed full-time.	1312	45.1
	Employed part-time	241	8.3
	Self-employed	363	12.5
	Unemployed	369	12.7
	Student	454	15.6
	Retired	67	2.3
	Long-term sick leave/medically retired	15	0.5
	Other	70	2.4
	Not answered	18	0.6
	Total	2909	100
**Which of these phrases would you say comes closest to your feelings about your income these days?**	Living really comfortably on present income	217	7.5
	Living comfortably on present income	621	21.3
	Neither comfortable nor struggling on present income	1268	43.6
	Struggling on present income	524	18.0
	"Really struggling on present income	254	8.7
	Not answered	25	0.9
	Total	2909	100

Table S2. Sexual attraction, sexual identity, and outness.

**Sexual Attraction** [1]	*N*	%	**Sexual Identity** [2]	*N*	%	**Outness** [3]	*N*	%
Men	2868	82.2	Gay or homosexual	2099	72.2	All or almost all	489	17.0
Women	511	14.6	Bisexual	440	15.1	More than half	643	22.4
Non-binary [4]	99	2.8	Straight or heterosexual	44	1.5	Less than half	423	14.7
I'm not attracted to anyone	5	0.1	I don't usually use a term	11	0.4	Few	941	32.7
Not answered	8	0.2	Not answered	314	10.8	None	371	12.9
						Not answered	9	0.3
Total	3491	100		2909	100		2876	100

[1] Who are you sexually attracted to?—Participants could select as many as apply. [2] Which of the following options best describes how you think of yourself? [3] Thinking about all the people who know you (including family, friends, and work or study colleagues), what proportion know that you are attracted to men?—Question shown when answer to question "who are you sexually attracted to?" was any of men. [4] People who identify as neither men nor women, or as both.

Table S3: Percentages of current partnerships, type of partnership, and when the last steady partnership broke up.

Do you currently have a steady partner?	*N*	%		*N*	%
No, I am single	1750	60.2	**When did your most recent steady relationship break-up?**		
			I've never had a steady relationship	450	15.5
			Within the last 24 hours	2	0.1
			Within the last 7 days	11	0.4
			Within the last 4 weeks	53	1.8
			Within the last 6 months	201	6.9
			Within the last 12 months	229	7.8
			Within the last 5 years	495	17.0
			More than 5 years ago	228	7.8
			Not answered	83	2.9
			Total	1752	100
Yes, I have a steady partner	867	29.8	**Are you currently in a steady relationship with** [1]		
			One man only	704	81.0
			More than one man (no women/non-binary)	63	7.2
			One non-binary only	4	0.5
			One woman	18	2.1
			More than one woman	10	1.2
			Not answered	3	0.3
I'm not sure or it's complicated	290	10.0	Total	869	100
Not answered	2	0.1			
Total	2909	100			

[1] Participants could select as many as apply.

Table S4. Buying and selling sex.

	When was the last time you paid a man to have sex with you?		When was the last time you were paid by a man to have sex with him?	
	N	%	N	%
Never	2224	76.5	2484	85.4
Within 24 hours	5	0.2	10	0.3
Within 7 days	44	1.5	17	0.6
Within 4 weeks	55	1.9	19	0.7
Within 6 months	117	4.0	35	1.2
Within 12 months	90	3.1	29	1.0
Within 5 years	146	5.0	90	3.1
More than 5 years ago	133	4.6	128	4.4
Not answered	95	3.3	97	3.3
Total	2909	100	2909	100

Table S5. Mental health. Men were asked to report how often they had been bothered by the following problems over the last two weeks (*n* = 2909).

	N (%)				
	**Not at All**	**Some Days**	**More than Half the Days**	**Nearly Every Day**	**Not Answered**
**Feeling nervous, anxious or on edge**	660 (22.7)	1397 (48.0)	417 (14.3)	420 (14.4)	15 (0.5)
**Not being able to stop or control worrying**	1361 (46.8)	1049 (36.1)	268 (9.2)	200 (6.9)	31 (1.1)
**Little interest or pleasure in doing things**	1084 (37.3)	1180 (40.6)	356 (12.2)	261 (9.0)	28 (1.0)
**Feeling down, depressed, or hopeless**	1137	1183	316	246	27

	(39.1)	(40.7)	(10.9)	(8.5)	(0.9)
	**Normal**	**Mild**	**Moderate**	**Severe**	**Not Answered**
**Anxiety and depression (PHQ-4) score (PHQ-4 score)** [1]	1116<br>(38.4)	1055<br>(36.3)	420<br>(14.4)	259<br>(8.9)	59<br>(2.0)

[1] Patient Health Questionaire-4 (PHQ-4) [2].

Table S6. Alcohol dependency.

	N (%)		
	**Yes**	**No**	**Not answered**
**Have you tried to cut down on your drinking?**	602<br>(23.3)	1952<br>(76.7)	26<br>(1.0)
**Have people annoyed you by criticising your drinking?**	186<br>(7.1)	2357<br>(91.4)	37<br>(1.4)
**Have you felt bad or guilty about your drinking?**	319<br>(12.4)	2222<br>(86.1)	39<br>(1.5)
**Have you taken a drink first thing in the morning to steady your nerves or get rid of a hangover?**	94<br>(3.6)	2458<br>(95.3)	28<br>(1.1)
**Alcohol dependency (CAGE4)** [1]	289<br>(9.9)	2560<br>(88.0)	60<br>(2.1)

[1] CAGE4 questions for alcohol use [1].

Table S7. Sex with non-steady male partners and condom use in the last 12 months.

	N	%		*N*	%
**Have you ever had any kind of sex with a man (please include any sexual contact, not just intercourse)?**			**Have you ever had intercourse with a man either with or without a condom?**		
No	85	2,9		212	7.3
Yes	2.821	97,0		2.603	89.5
Not answered	3	0,1		9	0.3
Total	2909	100		2909	100
**Have you had any kind of sex with a non-steady male partner?**			**How often were condoms used when you had intercourse with non-steady male partners?**		
No	742	25.5	Never	79	4.2
Yes	2153	74.0	Seldom	74	3.9
Not answered	14	0.5	Sometimes	132	7.0
			Mostly	540	28.6
			Always	1058	56.0
			Not answered	6	0.3
Total	2909	100	Total	1889	100
**Number of non-steady male**					
**Intercourse partners**			**Condomless intercourse partners**		
None	245	11.4		946	50.1
1	294	13.7		322	17.1
2	274	12.7		182	9.6
3	180	8.4		82	4.3
4	137	6.4		64	3.4
5	163	7.6		30	1.6
6	70	3.3		21	1.1
7	44	2.0		18	1.0
8	55	2.6		5	0.3
9	18	0.8		6	0.3
10	133	6.2		33	1.8
11–20	254	11.8		48	2.5
21–30	108	5.0		26	1.4
31–40	49	2.3		9	0.5
41–50	32	1.5		10	0.5

>50	78	3.6	19	1.0
Not answered	19	0.9	68	3.6
Total	2153	100	1889	100

Table S8. Sex under intoxication.

		N	%
**In the last 12 months, how much of the sex you've had with men has been under the influence of alcohol or any other drug?**	None of it	1573	58.6
	Almost none of it	582	21.7
	Less than half	256	9.5
	About half	82	3.1
	More than half	76	2.8
	Almost all of it	73	2.7
	All of it	29	1.1
	Not answered	13	0.5
	Total	2688	100

Table S9. Self-efficacy measures considering sex (n = 2909).

	***N* (%)**					
	**Strongly Disagree**	**Disagree**	**Not Sure**	**Agree**	**Strongly Agree**	**Not Answered**
**The sex I have is always as safe as I want it to be.**	60 (2.1)	184 (6.3)	417 (14.3)	1302 (44.8)	938 (32.2)	8 (0.3)
**I find it easy to say 'no' to sex I don't want.**	31 (1.1)	113 (3.9)	328 (11.3)	1084 (37.3)	1337 (46.0)	16 (0.5)

Table S10. HIV transmission, testing, and treatment knowledge (*n* = 2909). All the following statements are true.

	N (%)					
General knowledge	I knew this already	I wasn't sure about this	I didn't know this already	I don't understand this	I do not believe this	Not answered
AIDS is caused by a virus called HIV.	2835 (97.5)	47 (1.6)	19 (0.7)	2 (0.1)	4 (0.1)	2 (0.1)
You cannot be confident about whether someone has HIV or not from their appearance.	2706 (93.0)	116 (4.0)	35 (1.2)	23 (0.8)	25 (0.9)	4 (0.1)
Transmission						
HIV cannot be passed during kissing, including deep kissing, because saliva does not transmit HIV.	2581 (88.7)	229 (7.9)	53 (1.8)	1 (0.0)	27 (0.9)	18 (0.6)
You can pick up HIV through your penis while being 'active' in anal or vaginal sex without a condom, even if you don't ejaculate.	2479 (85.2)	293 (10.1)	107 (3.7)	0 (0.0)	9 (0.3)	21 (0.7)
You can pick up HIV through your rectum or vagina while being 'passive' during sex.	2788 (95.8)	55 (1.9)	36 (1.2)	2 (0.1)	2 (0.1)	26 (0.9)
Test						
There is a medical test that can show whether or not you have HIV.	2858 (92.3)	26 (0.9)	13 (0.5)	3 (0.1)	3 (0.1)	6 (0.2)
If someone becomes infected with HIV it may take several weeks before it can be detected in a test.	2629 (90.4)	171 (5.9)	93 (3.2)	4 (0.1)	8 (0.3)	4 0.1)
Treatment						
There is currently no cure for HIV infection.	2379 (81.8)	294 (10.1)	97 (3.3)	16 (0.6)	107 (3.7)	16 (0.6)
HIV infection can be controlled with medicines so that its impact on health is much less.	2689 (92.4)	135 (4.6)	53 (1.8)	2 (0.1)	6 (0.2)	24 (0.8)

**A person with HIV who is on effective treatment (called 'undetectable viral load') cannot pass his/her virus to someone else during sex.**	1466 (50.4)	709 (24.4)	495 (17.0)	36 (1.2)	187 (6.4)	16 (0.6)

Table S11. Post-exposure prophylaxis (PEP) and pre-exposure prophylaxis (PrEP) knowledge (*n* = 2909). All the following statements are true.

	N (%)					
	**I knew this already**	**I wasn't sure about this**	**I didn't know this already**	**I don't understand this**	**I do not believe this**	**Not answered**
**Post-exposure prophylaxis (PEP) attempts to stop HIV infection taking place after a person is exposed to the virus (for example by having intercourse without a condom).**	1640 (56.4)	387 (13.3)	814 (28.0)	34 (1.2)	24 (0.8)	10 (0.3)
**PEP is a one-month course of anti-HIV drugs.**	1158 (39.8)	505 (17.4)	1215 (41.8)	12 (0.4)	11 (0.4)	8 (0.3)
**PEP should be started as soon as possible after exposure, preferably within hours.**	1556 (53.5)	309 (10.6)	1006 (34.6)	5 (0.2)	8 (0.3)	25 (0.9)
**Pre-exposure prophylaxis (PrEP) involves someone who does not have HIV taking pills before as well as after sex to prevent him/her from getting HIV.**	1267 (43.6)	365 (12.6)	1209 (41.6)	36 (1.2)	19 (0.7)	13 (0.5)
**PrEP can be taken as a single daily pill if someone does not know in advance when they will have sex.**	963 (33.1)	416 (14.3)	1454 (50.0)	28 (1.0)	30 (1.0)	18 (0.6)
**If someone knows in advance when they will have sex, PrEP needs to be taken as a double dose approximately 24 hours before sex and then at both 24 and 48 hours after the double dose.**	379 (13.0)	376 (12.9)	2.043 (70.2)	47 (1.6)	32 (1.1)	32 (1.1)

Table S12. Hepatitis knowledge and knowledge of where to get hepatitis A and B vaccinations among men who could benefit from hepatitis A and B vaccine (i.e., men who had not been vaccinated against hepatitis A/B or who had not completed the course of vaccinations, or who did not know their hepatitis A/B vaccination status).

	N (%)					
	**I knew this already**	**I wasn't sure about this**	**I didn't know this already**	**I don't understand this**	**I do not believe this**	**Not answered**
**Hepatitis is an inflammation of the liver.**	2224 (76.5)	401 (13.8)	266 (9.1)	3 (0.1)	4 (0.1)	11 (0.4)
**Most cases of hepatitis are caused by viruses.**	2073 (71.3)	550 (18.9)	259 (8.9)	3 (0.1)	6 (0.2)	18 (0.6)
**There are several types of hepatitis viruses, named after the letters of the alphabet.**	2589 (89.0)	128 (4.4)	165 (5.7)	2 (0.1)	1 (0.0)	24 (0.8)
**Vaccines exist for both hepatitis A and hepatitis B.**	2457 (84.5)	329 (11.3)	101 (3.5)	2 (0.1)	2 (0.1)	18 (0.6)
**Doctors recommend men-who-have-sex with- men are vaccinated against both hepatitis A and hepatitis B viruses.**	1501 (51.6)	603 (20.7)	774 (26.6)	7 (0.2)	10 (0.3)	14 (0.5)
		**Hepatitis A**			**Hepatitis B**	
**Do you know where you could get vaccinated against**	No	671	40.6		620	40.8
	Yes	600	36.3		551	36.2
	Not sure	360	21.8		345	22.7
	Not answered	20	1.2		5	0.3
	Total	1651	100		1521	100

Table S13. Homophobic abuse: intimidation, insults, and violence (*n* = 2909).

When was the last time you		*N*	%
were stared at or intimidated because someone knew or presumed you are attracted to men?	Never	1316	45.2
	Within the last 24 hours	58	2.0
	Within the last 7 days	136	4.7
	Within the last 4 weeks	129	4.4
	Within the last 6 months	204	7.0
	Within the last 12 months	162	5.6
	Within the last 5 years	322	11.1
	More than 5 years ago	573	19.7
	Not answered	9	0.3
had verbal insults directed at you, because someone knew or presumed you are attracted to men?	Never	1298	44.6
	Within the last 24 hours	25	0.9
	Within the last 7 days	45	1.6
	Within the last 4 weeks	79	2.7
	Within the last 6 months	155	5.3
	Within the last 12 months	129	4.4
	Within the last 5 years	407	14.0
	More than 5 years ago	757	26.0
	Not answered	14	0.5
were punched, hit, kicked, or beaten because someone knew or presumed you are attracted to men?	Never	2553	87.8
	Within the last 24 hours	5	0.2
	Within the last 7 days	3	0.1
	Within the last 4 weeks	9	0.3
	Within the last 6 months	10	0.3
	Within the last 12 months	19	0.7
	Within the last 5 years	53	1.8
	More than 5 years ago	247	8.5
	Not answered	10	0.3

Table S14. Sources of condoms in the last 12 months and most common source.

	N	%
**Where have you got condoms from…?** [1]		
From an online pharmacy	119	2.9
Bought at a physical shop (not online)	2072	49.9
Bought from a vending machine	76	1.8
Free from clinics	265	6.4
Free from gay bars or clubs	232	5.6
Free from saunas	206	5.0
Free from gay or HIV community organisations	419	10.1
From friends or sex partners	376	9.0
Other answers	80	1.9
I have not got condoms in the last 12 months	299	7.2
Not answered	11	0.3
Total	4155	100

[1] Participants could select as many as apply.

Table S15. Recency of seeing/hearing any information about HIV or STIs specifically for men who have sex with men.

		N	%
**When was the last time you saw or heard any information about HIV or STIs specifically for men who have sex with men?)**	Never	64	2.2
	Within the last 24 hours	324	11.1
	Within the last 7 days	564	19.4
	Within the last 4 weeks	484	16.6
	Within the last 6 months	514	17.7
	Within the last 12 months	234	8.0
	Within the last 5 years	118	4.1
	More than 5 years ago	71	2.4
	Not answered	536	18.4
	Total	2909	100

Table S16. Satisfaction with support and information received during HIV testing.

	**HIV positive**		**HIV negative** [1]	
**When you were diagnosed HIV positive/tested for HIV, how satisfied were you with the support and information you received?**	*N*	%	N	%
Did not receive any support or information	43	13.5	620	33.0
Very satisfied	88	27.6	677	36.1
Satisfied	109	34.2	396	21.1
Dissatisfied	41	12.9	37	2.0
Very dissatisfied	25	7.8	17	0.9
I don't remember/I did not think about it	13	4.1	100	55.3
Not answered			31	1.7
Total	319	100	1878	100

[1] Participants tested for HIV and diagnosed HIV-negative.

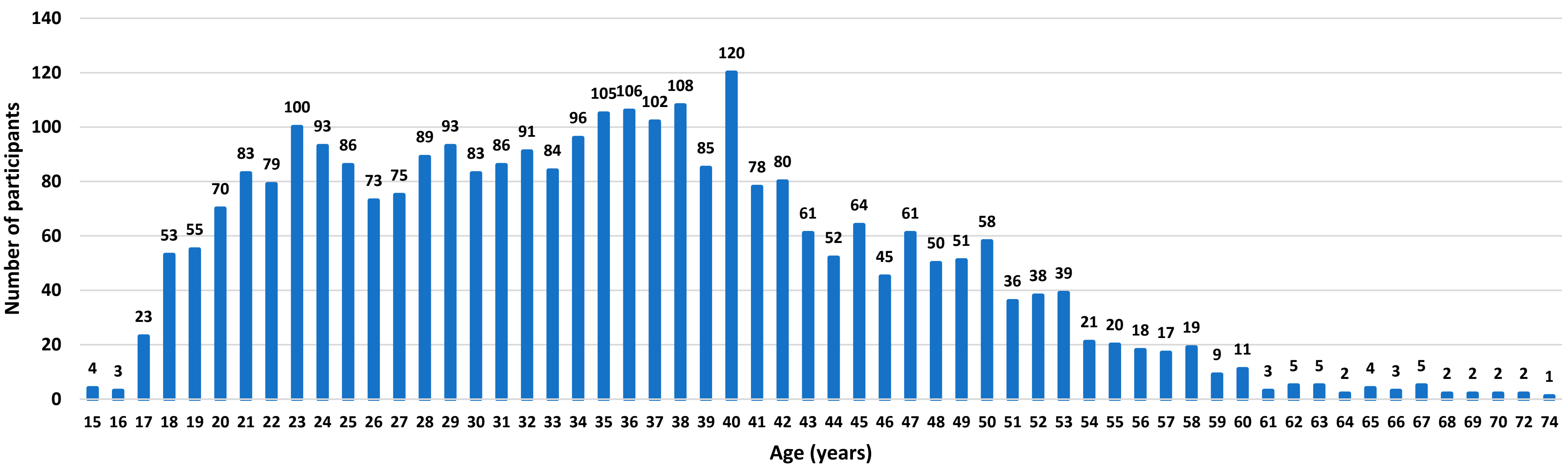

Figure S1. Age in years in the EMIS-2017 sample in Greece (*N* = 2909).

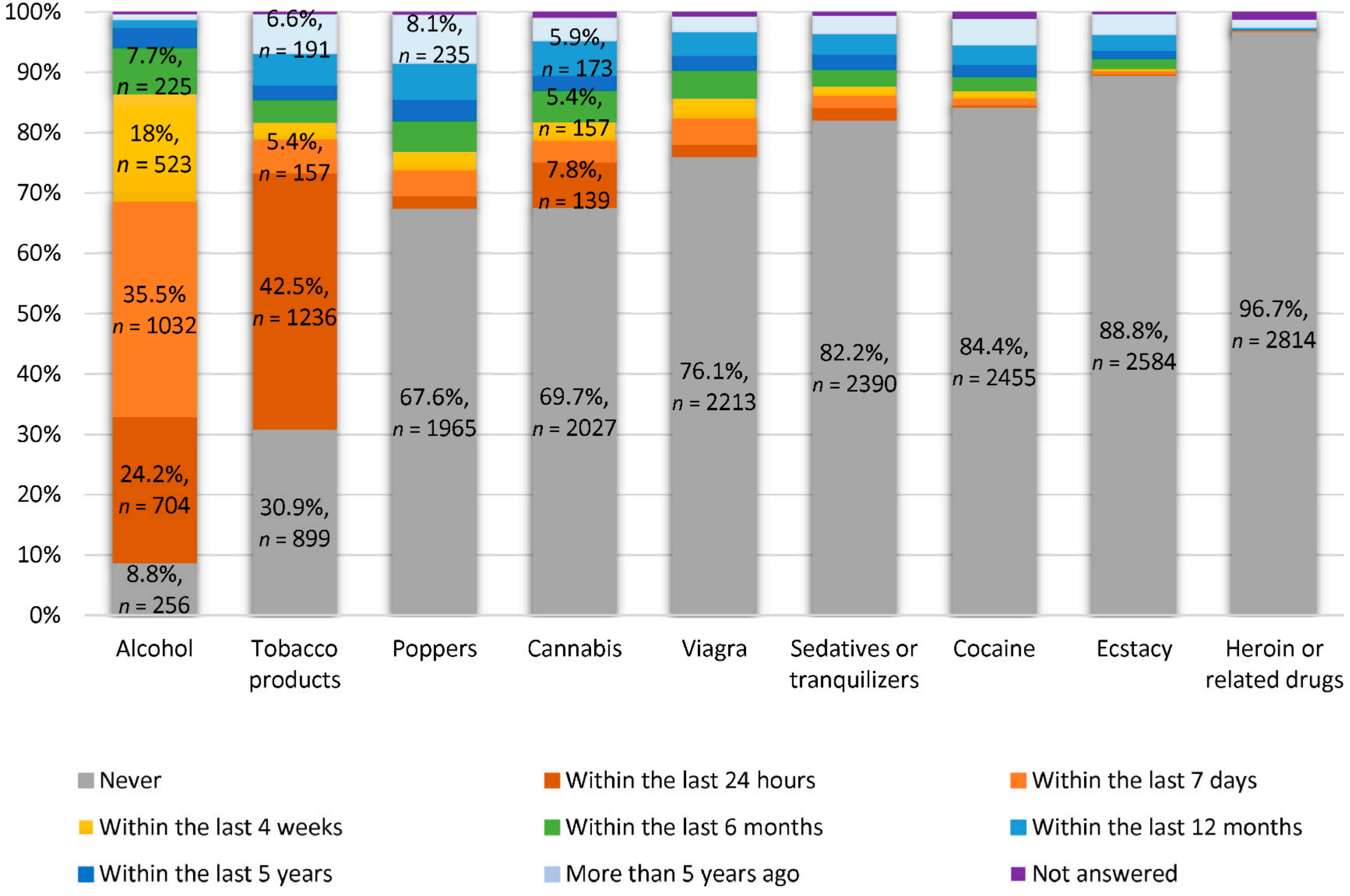

Figure S2. Recency of using selected substances. Not included are synthetic cannabinoids, amphetamine, crystal methamphetamine, mephedrone, synthetic stimulants other than mephedrone, GHB/GBL, ketamine, LSD, crack cocaine.
